# The Efficacy of Tranexamic Acid in Reducing Perioperative Drainage in Cardiac Surgery with Cardiopulmonary Bypass

**DOI:** 10.21470/1678-9741-2023-0181

**Published:** 2024-04-15

**Authors:** Murat Ziya Bağiş, Bişar Amaç

**Affiliations:** 1 Clinic of Cardiovascular Surgery, Health Sciences University, Sanliurfa Mehmet Akif Inan Training and Research Hospital, Sanliurfa, Turkey; 2 Department of Perfusion, Health Sciences University, Sanliurfa Mehmet Akif Inan Training and Research Hospital, Sanliurfa, Turkey

**Keywords:** Coronary Artery Bypass, Heart-Lung Machine, Tranexamic Acid, Bleeding, Drainage

## Abstract

**Introduction:**

Although cardiopulmonary bypass procedures remain a critical treatment option for heart disease, they come with risks, including hemorrhage. Tranexamic acid is known to reduce morbidity and mortality in surgical hemorrhage.

**Objective:**

This study aimed to evaluate the efficacy of tranexamic acid, which is routinely used to treat hemorrhage, in decreasing the amount of intraoperative and postoperative drainage.

**Method:**

A total of 80 patients who underwent cardiac surgery with cardiopulmonary bypass were included in this retrospective study. Forty patients who received tranexamic acid during the operation were assigned to Group 1, while 40 patients who did not receive tranexamic acid were assigned to Group 2. Patient data were collected from the hospital computer system and/or archive records after applying exclusion criteria, and the data were recorded. Statistical analyses were then performed to compare the data.

**Results:**

Age, sex, height, weight, body surface area, flow, and ejection fraction percentages, preoperative hematological parameters, and intraoperative variables (except tranexamic acid) were similar between the groups (P>0.05). However, there were statistically significant differences between the groups in terms of intraoperative (through the heart-lung machine) and postoperative red blood cell transfusion rates, intraoperative and postoperative bleeding drainage amounts, as well as postoperative hematocrit, hemoglobin, platelet, and red blood cell levels (P<0.05).

**Conclusion:**

We concluded that intraoperative and postoperative use of tranexamic acid in patients who underwent coronary artery bypass grafting with cardiopulmonary bypass has positive effects on hematological parameters, reducing blood product use, and bleeding drainage amount.

## INTRODUCTION

**Table:** 

Abbreviations, Acronyms & Symbols	
ACC	= Aortic cross-clamping
BSA	= Body surface area
CABG	= Coronary artery bypass grafting
CPB	= Cardiopulmonary bypass
EF	= Ejection fraction
HCT	= Hematocrit
HGB	= Hemoglobin
HLM	= Heart-lung machine
PLT	= Platelet
RBC	= Red blood cell
RBCS	= Red blood cell suspension
SD	= Standard deviation
TXA	= Tranexamic acid

Today, cardiovascular diseases, such as coronary heart disease, are a leading cause of death worldwide. Coronary artery bypass grafting (CABG) is a standard of care for those with coronary artery disease^[1]^. Improvements in cardiopulmonary bypass (CPB) have enabled significant advances in cardiac surgery. CPB provides a bloodless field in cardiac surgery and is a necessary treatment modality in some cases. Despite the fact that the use of the CPB system facilitates surgery, it carries various risks^[2]^. While surgical techniques have improved with CPB, it is important to manage associated risks, including coagulopathic bleeding, which poses a significant risk during weaning from CPB and is associated with poor outcomes and mortality in cardiac surgery patients^[3]^.

The use of antifibrinolytics has been shown to reduce blood loss and subsequent transfusions, which are associated with major morbidity and mortality, in various surgical procedures such as cardiac surgery, trauma, orthopedic surgery, liver surgery and solid organ transplantation, obstetrics and gynecology, neurosurgery, and non-surgical diseases. The amount of evidence supporting the effectiveness of tranexamic acid (TXA), a synthetic lysine analog, has grown over time. It was first patented in 1957, and its use has gradually increased over the years^[4]^.

TXA has gained significant attention due to its potential to reduce morbidity and mortality in surgical and traumatic bleeding. Studies have shown that it can reduce mortality from traumatic bleeding by one-third without causing significant safety concerns. In patients with surgical bleeding that require transfusions, intravenous TXA has been shown to reduce blood loss. It can also be used topically to reduce bleeding. Its efficacy is being further investigated in large pragmatic studies regarding traumatic head injuries, postpartum hemorrhage, and upper gastrointestinal bleeding. Adverse effects of TXA, which interfere with cerebral gamma aminobutyric acid and glycine receptors, are rare, except at high doses where neurological events have been recorded. Furthermore, clinical studies indicate that using a higher dose does not increase efficacy; namely, a dose of 1 g of TXA administered intravenously to an adult patient has maximum efficacy without increasing adverse effects^[5]^.

TXA provides a beneficial treatment for bleeding in tissues rich in plasminogen activators or under endocrine influences, as well as in cases where there is a risk of secondary hemorrhage due to local or generalized primary hyperfibrinolysis following trauma. Additionally, it is used in reducing hyperfibrinolytic bleeding in cardiovascular surgeries and has a dose-independent effect on fibrinolysis parameters. Since its introduction in 1962, TXA has become a major antifibrinolytic agent for managing hemorrhage in patients undergoing cardiac surgery, particularly after the discontinuation of aprotinin in 2007^[6-8]^.

This prospective study aimed to investigate the efficacy of TXA, a commonly used antifibrinolytic agent, in reducing bleeding for both intraoperative and postoperative hemorrhage. For this purpose, the study compared perioperative bleeding rates between two patient groups - those who received TXA and those who did not - during cardiac surgery with CPB.

## METHODS

### Type of Study

This clinical study is a prospective cohort study.

### Ethical Approval

The study received approval from the Harran University Clinical Research Ethics Committee prior to initiation (approval no.: HRU/23.02.06, date: 23.01.2023). All participants provided written informed consent before undergoing surgery and were informed about the rationale and design of the study. The study was conducted in accordance with the principles outlined in the Helsinki Declaration.

### Population of the Study and Creation of the Groups

This prospective study included 80 patients who underwent cardiac surgery with CPB. The patients were divided into two groups: Group 1 (TXA group), which consisted of 40 patients who received TXA during the operation (intraoperative), and Group 2 (no TXA group), which consisted of 40 patients who did not receive TXA.

### Data Collection

After applying the exclusion criteria, the data of the patients who underwent cardiac surgery with CPB were recorded as preoperative, intraoperative, and postoperative. Descriptive data such as age, sex, height, weight, body surface area (BSA), flow, and ejection fraction (EF) percentage were collected. Intraoperative data included aortic cross-clamping time, total perfusion time, TXA dose rates, and type of surgery performed (CABG numbers). The preoperative and postoperative hematological variables (hematocrit, hemoglobin, platelet, and red blood cell) and perioperative variables of the groups were also recorded, including red blood cell suspension (RBCS) transfusion (perioperative), perioperative drainage amount, and postoperative drainage amount.

### Inclusion and Exclusion Criteria

Patients between the ages of 20 and 85 years who consecutively underwent CABG with CPB operation in our clinic between 01.01.2021 and 31.12.2022 were included in the study after applying exclusion criteria. Patients who received preoperative anticoagulant drugs, underwent emergency cardiac surgery, required additional cardiac surgery such as an aortic aneurysm or dissection, had known systemic inflammatory disease, underwent cardiac surgery reoperation, were on chronic hemodialysis, had hematologic disease or an active intravascular coagulation disorder (such as pulmonary embolism, deep vein thrombosis, antithrombin III deficiency, or arterial thrombosis), had a history of thrombophilia, or had an allergy to intravenous TXA or its active ingredient were excluded from the study.

### Surgical Procedure

Standard surgical techniques were used. After midline sternotomy, arterial cannulation was performed through the ascending aorta and venous cannulation was performed through the right atrium with a two-stage venous cannula. Left mammary artery graft was used in all cases. Saphenous vein was applied to other coronary grafts. All patients underwent complete revascularization.

The blood flow rates during the extracorporeal circulation of the patients included in the study were determined according to their BSA (2.4 L/min/m2). Oxygenator and tubing set suitable for the weight of the patient and cannula diameters suitable for BSAs were used. Membrane oxygenator/tubing sets with integrated arterial filters were used. Tubing set was used as 1/2 venous line diameter and 3/8 arterial line diameter. All patients underwent 32 °C hypothermia during extracorporeal circulation. Arterial line pressures were maintained on average between 150 and 180 mmHg during CPB. Active coagulation time was kept ≥ 480 seconds by providing adequate anticoagulation (400 U/kg). As a prime solution, 1200 mL of balanced solution (Isolayte), 150 mL of 20% mannitol, 5,000 units of heparin, and 2 g of cefazolin were used. In patients for whom isothermal blood cardioplegia solution was administered (32 °C), initially, the amount of cardioplegia solution was administered as kg × 15 mL (full dose), and the additional maintenance dose applied every 20 minutes was administered as a half dose (1/2). As per cardioplegia content, it was prepared by adding potassium chloride, magnesium, and sodium bicarbonate to oxygenated patient blood taken from CPB equipment. After weaning from CPB, 1 mg of protamine sulfate was administered for every 100 mg of heparin applied to neutralize the effect of heparin.

### Tranexamic Acid Protocol

The administration of TXA included three doses, as follows.

1. TXA anesthesia infusion dose: this dose was administered as an infusion of 15 mg/kg in 100 cc saline over 20 minutes through the central venous catheter following anesthesia induction.
2. TXA prime dose: an additional dose of 1mg/kg was added to the heart-lung machine prime solution.
3. TXA heart-lung machine infusion dose: this dose was administered as an infusion into the extracorporeal circulation equipment of 8 mg/kg in 100 cc saline over 60 minutes while the patient was connected to the heart-lung machine.

### Statistical Analysis

The statistical analyses were performed using SPSS Inc. Released 2007, SPSS for Windows, version 16.0, Chicago: SPSS Inc. computer software. Means and standard deviations were calculated for continuous and ordinal data. The normality distribution was assessed using the Kolmogorov-Smirnov and Shapiro-Wilk tests. The Student’s *t*-test and Mann-Whitney-U tests were used for normally and non-normally distributed data, respectively. Nominal data were analyzed using frequency and percentage, and the Chi-square test was used for comparison. A *P*-value < 0.05 was considered statistically significant.

## RESULTS

As seen in Table 1, there were no significant differences with regard to age, sex, height, weight, BSA, flow, and EF percentage between the two groups (*P*>0.05). As seen in Table 2, the number of CABG performed in the groups was comparable, with no statistically significant difference (*P*>0.05). Table 3 shows the minimum, maximum, mean, and standard deviation values of the TXA applied group.

**Table 1:** Demographic and descriptive data of groups.

	Group 1 - received TXA (n=40)	Group 2 - did not receive TXA (n=40)	*P*-value
	Mean ± SD	Mean ± SD	
Age (years)	61.15 ± 10.00	62.40 ± 9.20	0.667
Sex (female)	n=16 (40%)	n=17 (42.4%)	0.658
Height (cm)	164.15 ± 10.73	164.82 ± 10.44	0.698
Weight (kg)	78.72 ± 13.53	76.50 ± 13.60	0.856
BSA (m^2^)	1.87 ± 0.17	1.85 ± 0.18	0.682
Flow (lt)	4.41 ± 0.55	4.36 ± 0.55	0.836
EF (%)	51.15 ± 8.71	50.12 ± 9.48	0.816

BSA=body surface area; EF=ejection fraction; SD=standard deviation; TXA=tranexamic acid

**Table 2:** Number of CABG performed in groups.

	Group 1 - received TXA (n=40)		Group 2 - did not received TXA (n=40)		*P*-value
	Frequency (n)	Percentage (%)	Frequency (n)	Percentage (%)	
CABG 2	5	12.5	3	7.5	0.443
CABG 3	16	40.0	18	45.0	
CABG 4	13	32.5	14	35.0	
CABG 5	6	15.0	5	12.5	
Total	40	100.0	40	100.0	

CABG=coronary artery bypass grafting; TXA=tranexamic acid

**Table 3:** Group 1 - TXA administration data.

Group 1 - TXA (n=40)					
	N	Minimum	Maximum	Mean	SD
TXA anesthesia infusion^*^, (mg)	40	720.00	1800.00	1181.00	205.82
TXA prime^**^, (mg)	40	48.00	120.00	78.65	13.514
TXA HLM infusion^***^, (mg)	40	384.00	960.00	630.00	110.11
Valid n (listwise)	40				

HLM=heart-lung machine; SD=standard deviation; TXA=tranexamic acid

* TXA dose administered as an infusion in 100 cc of physiological saline at 15 min. from the central venous catheter after anesthesia induction

** TXA dose added to HLM prime solution

*** TXA dose administered as an infusion to extracorporeal circulatory equipment in 100 cc of physiological saline at 60 min. while the patient is connected to the HLM

**Fig. 1:**
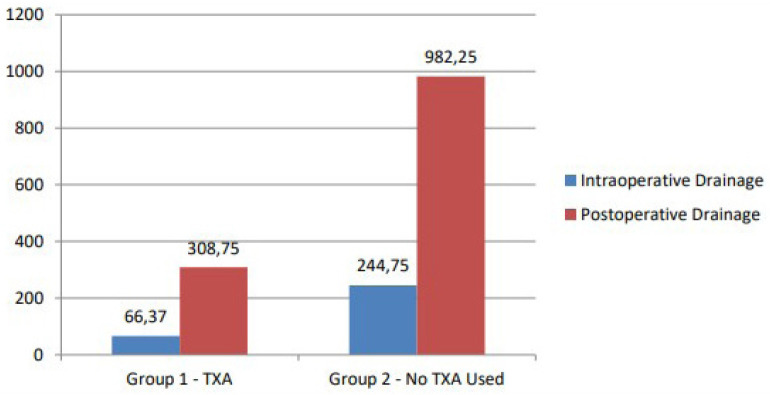
Comparison of the drainage amount rates between the groups. TXA=tranexamic acid.

As seen in Table 4, there were no significant differences between the groups in terms of total perfusion time, aortic cross-clamping time, preoperative hematocrit, hemoglobin, platelet, and red blood cell levels (*P*<0.05). Nonetheless, there were significant differences between the groups in terms of intraoperative (through the heart-lung machine) and postoperative red blood cell transfusion rates, intraoperative and postoperative bleeding drainage amounts (Figure 1), and postoperative hematocrit, hemoglobin, platelet, and red blood cell levels (*P*<0.05).

**Table 4:** Comparison of preoperative variables between the groups.

	Group 1 - received TXA	Group 2 - did not receive TXA	*P*-value
	(n=40)	(n=40)	
	Mean ± SD	Mean ± SD	
Total perfusion time (min.)	95.97 ± 38.05	99.72 ± 38.95	0.675
ACC time (min.)	52.90 ± 21.68	61.00 ± 28.89	0.104
Heart-lung machine RBCS (unit)	0.62 ± 0.89	2.15 ± 0.66	0.016
Postoperative RBCS (unit)	0.67 ± 0.828	3.22 ± 1.09	0.028
Intraoperative drainage (mL)	66.37 ± 35.48	244.75 ± 96.20	0.000
Postoperative drainage (mL)	308.75 ± 132.45	982.25 ± 226.95	0.006
Preoperative HCT (%)	40.54 ± 5.00	39.24 ± 4.20	0.061
Preoperative HGB (g/dL)	13.70 ± 1.85	13.75 ± 1.63	0.330
Preoperative PLT (103/µL)	247.28 ± 52.75	238.28 ± 51.23	0.855
Preoperative RBC (mcL)	4.65 ± 0.69	4.51 ± 0.62	0.310
Postoperative HCT (%)	28.49 ± 4.65	25.31 ± 2.99	0.044
Postoperative HGB (g/dL)	9.96 ± 1.45	7.67 ± 2.08	0.046
Postoperative PLT (103/µL)	200.22 ± 59.56	187.72 ± 30.73	0.000
Postoperative RBC (mcL)	3.30 ± 0.52	3.07 ± 0.36	0.038

ACC=aortic cross-clamping; HCT=hematocrit; HGB=hemoglobin; PLT=platelet; RBC=red blood cell; RBCS=red blood cell suspension; SD=standard deviation; TXA=tranexamic acid

## DISCUSSION

Coronary artery disease is the leading cause of death in developed countries^[1]^. CABG operations with CPB remain an important treatment method and sometimes the only method. The aim of this study was to evaluate the efficacy of TXA, a drug commonly used to control bleeding, on the amount of bleeding drainage during and after cardiac surgery. The study revealed that the use of TXA had positive effects on intraoperative and postoperative hematological parameters, reducing blood product use, and the amount of bleeding drainage in patients who underwent CABG with CPB. Besides, demographic data and intraoperative surgical variables, except for the use of TXA, were similar between the groups and did not affect the results.

Rostami et al.^[1]^ conducted a study to investigate the effect of TXA in reducing postoperative bleeding in patients undergoing CABG. They randomly divided 62 patients into two groups: the TXA group and the control group. After separation from the heart-lung machine, 2 g of TXA was injected locally into the mediastinum in the TXA group, while the control group was given the same amount of normal saline (100 cc). Significant differences were found between the two groups in terms of postoperative bleeding, hematocrit, platelet transfusion, operative time, and fresh frozen plasma received (*P*=0.0001; *P*=0.01; *P*=0.0001; *P*=0.0001; *P*=0.0001, respectively), with lower requirements observed in the TXA group^[1]^. These results are consistent with our study, which showed that in the TXA group, intraoperative (through the heart-lung machine) and postoperative red blood cell transfusion rates (0.62 ± 0.89 and 0.67 ± 0.828, respectively), and intraoperative and postoperative bleeding drainage amounts (66.37 ± 35.48 and 308.75 ± 132.45, respectively) were lower, and postoperative hematocrit (28.49 ± 4.65), hemoglobin (9.96 ± 1.45), platelet (200.22 ± 59.56), and red blood cell (3.30 ± 0.52) levels were higher.

Similarly, Myles et al.^[9]^ found that TXA use in patients undergoing coronary artery surgery was associated with a lower risk of bleeding within 30 days of surgery compared to the control group, without an increased risk of death or thrombotic complications. Several other studies have also reported that TXA use reduced postoperative drainage (bleeding)^[10-12]^. Chen et al.^[13]^ reported in their study that TXA use could reduce the requirement for blood products without increasing the risk of postoperative seizures after CPB.

Shi et al.^[14]^ conducted a study comparing the efficacy and side effects of high-dose and low-dose TXA in patients who underwent cardiac surgery with CPB. The high-dose group received a 30 mg/kg bolus, 2 mg/kg prime, and 16 mg/kg/hour maintenance dose (n=1525), while the low-dose group received a 10 mg/kg bolus, 1 mg/kg prime, and 2 mg/kg/hour maintenance dose (n=1506). The study found no significant difference in the need for red blood cell transfusion and adverse events (30-day mortality, seizures, renal dysfunction, and thrombotic events) between the high-dose and low-dose groups^[14]^. Besides, Murkin et al.^[15]^ reported that high-dose TXA use in cardiac surgery performed with CPB in elderly patients was associated with clinical seizures in susceptible patients^[15]^. There are also studies that have reported similar results^[16,17]^.

In their study, Guo et al.^[18]^ investigated the efficacy of different TXA regimens in reducing the requirement for transfusion in cardiac surgery. They concluded that TXA was effective in all types of cardiac surgeries, and the low-dose regimen was equally effective in reducing the transfusion rate without increasing the risk of seizures, making it the preferred option^[18]^. Similarly, in our study, we also found that low-dose TXA had a significant effect on reducing red blood cell transfusion rates, intraoperative and postoperative bleeding drainage amounts, and improving postoperative hematocrit, hemoglobin, platelet, and red blood cell levels. Therefore, we consider the use of low-dose TXA as a sufficient and effective option.

### Limitations

The limitations of our study include its single-center design and relatively small sample sizes. We recognize that conducting multicenter studies with more sample sizes would provide more comprehensive data on the use of TXA in cardiac surgery.

## CONCLUSION

Our study showed that administering TXA during CABG operations with CPB resulted in positive outcomes, including decreased need for RBCS transfusion from the heart-lung machine, reduced amount of intraoperative and postoperative bleeding drainage, and improved postoperative levels of hematocrit, hemoglobin, platelets, and red blood cells. Based on the results of our study, we conclude that the use of TXA has beneficial effects on hematological parameters, reducing blood product use and bleeding drainage in patients undergoing CABG surgery with CPB.
